# Relations of the Integumentary and Generative Organs

**Published:** 1873-03

**Authors:** James N. Hyde

**Affiliations:** Chicago


### ARTICLE V.—*Relations of the Integumentary and Generative Organs.* By JAMES N. HYDE, M.D., Chicago.

The exterior investment of the body, including its various glandular, hairy and ungual appendages, is manifestly designed to protect and preserve the structure which it envelops. Viewed in this light alone, it is not obvious that the cutaneous surface has either an anatomical, physiological or pathological connection with those organs which are concerned in the reproduction of the species. And yet it may be made to appear, from a collation and comparison of well-ascertained facts, that, not only does such a relation exist, but that it exceeds that acknowledged union between each organ of the body and every other, by virtue of which the whole enjoys a symmetrical existence.

In the human fœtus, the germinal membrane of the blastodermic vesicle, soon after the segmentation of the vitellus, subdivides into two layers, which rapidly assume distinctive characters, and eventually give rise to two widely different systems of the living economy. The serous or animal layer is that which is external, and from it is derived the integument, as well as the organs which are essential to animal life. The mucous or vegetative layer is internal, and from it are developed the true viscera and the organs necessary to the continuance of organic life. These two lives are, thus early in embryonic existence, distinctly separated; and, thereafter, as Bichat says: "they may exist, the one independent of the other, though they were, without doubt, intended to be reciprocally supplementary."

Since, then, there is found to be a common primitive source of derivation for each of two distinctly different systems in one individual, it is natural to conclude that we shall discover certain physiological and pathological relations between those organs which are engaged in the fulfillment of the functions of the same life. It has been already seen that the integument is a derivative from the external animal layer of the ovum; let us ask in which of the two lives are the reproductive organs most interested, and whether, either wholly or in part, they are descended from the same embryonic layer.

About the fifth or sixth week of fœtal life an external cloaca can be seen, which is the termination common to the intestines, the uterus and the sexual organs. By the tenth week, the "uro-genital sinus" appears, in consequence of the development of a transverse band separating it from the anus. This sinus subsequently, in consequence of a similar process of division, displays a "pars genitalis" and a "pars urinaria," the latter extending to the urachus. Two folds of integument on either side of the orifice develop into either scrotum or labia, with an erectile body between, which is either subsequently retracted to form a clitoris, or elongated to become a penis.

So much for the external genitals. They evidently originate from the same primitive layer as the other external portions of the fœtus. The uterus, far from being the result of a coalescence of the Fallopian tubes, as was at one time believed, is derived, with the vagina, from the genital portion of the "uro-genital sinus"; and the distinction between the two is secondary, and effected by a subsequent demarcation of that portion of the sinus into a vaginal and uterine tract.

But it is generally stated that the essential organs of reproduction are the testes and ovaria. These are, it is true, derived, like the viscera of digestion, from the internal vegetative layer of the blastoderm, but the subsequent career of each—of the testes, at least—differs widely from that of their congeners. They are first formed in a mass of blastema lying in immediate connection with the Wolffian bodies. But very early in fœtal existence, the gubernaculum is found starting from the filamentous tissue of the scrotum, and fixing itself to the antetype of the epididymis, just as the round ligament rises from the labium of the female and attaches itself to the ovary. The testes then commence their descent, and, at the completion of pregnancy, ordinarily arrive in the scrotum, the cavity of which is speedily cut off from the general abdominal interior. Thus the testes are by a natural process set aside, as it were, from the organs concerned in organic life, and placed in immediate connection with those external parts, with whose function they are so intimately concerned. The ovary, on the contrary, remains fixed in the abdominal cavity, and, so far as regards its location, is not to be distinguished from portions of the vegetative apparatus.

It should be stated, however, that, even so recently as 1872,* it has been announced that the "cord" or "duct of Miller," which extends into the pelvis from a point between the Wolffian duct and the generatic glands, forms, by subsequent union with its fellow, the uterus and vagina, the divergent portion being converted into the Fallopian tubes. In the female, atrophy of the Wolffian bodies is said to produce the parovarium, or organ of

* Dr. Jenks, Am. Journal of Obstetrics, p. 738.

Rosenmuller, and in the male, the epididymis; the Wolffian duct being subsequently converted into the vas deferens.

Now, if the ovary and its function be physiologically considered, it will be observed that in one important particular it differs from all other adjuncts of reproduction, and is assimilated to the organic viscera. The function of other accessories of generation may remain unexcited and inactive during the entire life of an animal or vegetable, without impairment or derangement of physical health. This is not true of ovulation.

"Generation," says Bichat, "does not enter into the series of phenomena incident to organic and animal life. These are essential to the individual—that to the species. Its functions are aroused, only when all others have long been exercised, and its career is at an end when the latter are still in full activity. In most animals its periods of excitement are separated by long intervals of nullity; in man, where such remittance is less prolonged, it has no greater connection with other functions. Ablation of the generative organs is usually succeeded by a general increase in the processes of nutrition. The eunuch possesses, it is true, less vital energy; but the general phenomena of life are, in his case, displayed with greater profusion." While this is true, it is clear that the two lives, the organic and the animal, are best studied in connection with the alternative phases of *asexual* life, and *sexual* life. That life may be termed asexual in the adult, where no communication of the sexes occurs, and in sexual life, strictly speaking, the functions of the generative organs are duly awakened to activity. Now in asexual life, ovulation, when no abnormal conditions exist, proceeds with undeviating regularity; and experience everywhere teaches that such regularity is essential to perfect organic life. Interference with ovulation disturbs the entire economy, but its effects are displayed to a greater degree by perversions of order in the vegetative processes, than in those pertaining to animal life. The hæmatopœtic function is, in such cases, almost primarily deranged.

Nor need it be concluded that the uterus, if it be a development from the external blastodermic layer of the germ, has more than a sympathetic connection with the process of ovulation. This is abundantly demonstrated by cases in which there is a congenital absence of the womb. In the October number of the "American

Journal of Medical Sciences," for 1872, Dr. T. R. Brown, of Baltimore, reports a case of vicarious menstruation by epistaxis, in which death occurred from causes unconnected with the peculiar malformation; and in which an autopsy was made. No uterus was discovered, the bladder and rectum were firmly adherent—the ovaries were perfectly formed and in a normal position. Ovulation had recently occurred, and corpora lutea were found in various stages of degeneration.

The development of the sexual organs of plants seems to indicate the natural plan of their morphology. The essential parts of a pistil are the ovary and the stigma—the style may be viewed as the prototype of a Fallopian tube. The stigma corresponds to the external genitals, the ovary to the gland called by the same name in animals. Now the simple pistil, according to the botanist, Gray, "consists of the blade of a leaf, curved until its margins meet, and forming a closed pod or case, which is the true ovary. The upper face of the leaf answers to the inner face of the ovary—the lower to its outer surface; the tapering summit rolled together and prolonged, forms the style, when there is any; and the edges of the altered leaf, turned outward, either at the tip or along the inner side of the style, form the stigma." But the function of the leaf is that of a true viscus of digestion. No other portion of a plant exhibits greater organic activity. It is a depository of food in the seed-leaf of the bean. In the Sarracenia Purpurea, (so much vaunted a few years since in the treatment of variola) it serves, like the stomach of the camel, as a water carrier. In the Dionea, it is a fly-trap; in the tulip, its underground base is a reservoir for the nutriment requisite during the ensuing year. This indicates the affinities of the ovary in vegetable life. Let us ask further, if ovulation is essential to asexual life in plants, and whether it occurs where there is a natural impediment to sexual union.

Monœcious plants, as is well known, produce perfect flowers and seeds when individuals are isolated by transplantation or otherwise. Of the Atralia Spinosa, a polygamous plant, Mr. Thomas Meehan, of the Academy of Natural Sciences in Philadelphia, says: "When the flower-scape elongates, a very strong umbel of female flowers appears. A secondary series of female flowers succeeds this, and finally, a third series appears, which become filled with polleniferous anthers. The whole of the first series of flowers falls unfertilized, *before the male ones can possibly appear.*"

To return to the ovary of the human female, it may be further observed, that, in an anatomical point of view also, it is distinguished from the other generative organs. The mucous lining of the urethra is continuous with that of the secreting surface of the testis. The mucous wall of the vagina and uterus, on the contrary, is not continuous with the interior of the ovary; but the Fallopian tubes open abruptly to the peritoneal cavity; and this is said by anatomists to be the sole instance, in the body, of continuity of a mucous and serous surface. Reichert held, that "in the grouping of tissues, as soon as one part can be made out to be continuously (by union, not mere juxtaposition) connected with another, both must be regarded as parts of a common whole." When, therefore, we do not find such continuity between parts whose function is correlated, it is natural to inquire into the reasons for departure from an established rule.

Certain pathological facts strengthen the idea that the ovary is removed in its diseases from the other generative organs. Metastasis of mumps, for example, occurs in the male, from the parotid gland to the testis; in the female, to the mamma and never to the ovary. Now the mammary gland is produced by a development from the external surface. Remak was the first to establish the fact that "glands in general must be regarded as consequent upon a direct process of proliferation on the part of epithelial structures." The mamma is formed by a repeated division of epithelial cells, until a little process extends inward, spreads out laterally, and finally constitutes a body continuous with originally external cells. The ovaries, as Virchow has pointed out, are the only exceptions to this law in the human body, since their follicles are only periodically open. But the mamma exhibits no functional activity in asexual life, while the function of the ovary is essential to perfect asexual life in the female.

The history of cancer also throws some light on this subject. Paget has shown that "cancer-cells are formed on the type of excretory gland cells and epidermal cells." The term "heterologous" cannot then be properly applied to pathological formations, if they be all greater or less imitations of simple tissue, occurring at unusual times, or in unusual locations, or assuming development of an usual grade. We should, therefore, consistently expect to find that cancer most frequently invades such glands and tissues as are found in the integumentary and generative systems. Such is, in fact, the case. The lip, the mamma, the skin, and the testicle, are exceedingly apt for the formation of primary cancerous growths. Scirrhous tumors of the skin occur both secondarily and primarily, and occasionally precede mammary cancer. The latter affection occurs in ninety-eight per cent. of cancerous females—and generally between the ages of 45 and 50 years—or about the critical period of the menopause. About thirty per cent. of cases of medullary cancer affect the testicle—a larger proportion than that applying to any other single organ. Cancer of the ovary is extremely rare, and, when it occurs, it is generally secondary to an affection of contiguous parts. On the other hand, it may be said, that the cystic degenerations of the ovary are equally rare in the other organs of generation. Yet Dr. Walshe, in an endeavor to dissociate the phenomena of cancerous growths from all sexual complications, observes, that "everything which has been said to explain the liability of the breast and the womb to this disease, may be equally said of the ovaries, which are comparatively rarely cancerous." The reasons which explain the want of affinity between these organs, have already been considered.

Comparative physiology establishes with great distinctness a relation between the integument and the sexual parts. It is unnecessary to enlarge upon the development of the antlers of the stag, the increase in brilliancy of the plumage of certain birds, and the changes in the color and character of the fur of several species of animals, during seasons of sexual excitement, in order to develop such a relation. Indeed, it seems as if the outgrowth of external genitals bore an inverse ratio to the exhibition of sexual difference in the integument, in some forms of the animal kingdom. In those, for example, which have external organs of copulation largely developed, difference of sex is less noticeable in the skin and its appendages; while in those whose reproductive organs are wholly internal, as in those birds which have a common duct for the extrusion of the semen, or ova, and fæces, difference of sex is strongly declared by feather-coloring, comb, wattles, talons, etc. Among marsupials, the "Macropus Giganteus," or kangaroo, has a common cloaca for intestinal and genito-urinary function, and the female is distinguished externally by the sac in which her young are carried.*

The changes coincident with the arrival of puberty in man, are marked externally by corresponding alterations. In the male, the beard appears, and the pubic and axillary hairs. In the female, there is a notable difference in the facial color and expression. Aberrations from normal pubescence are immediately indicated upon the surface of the body. "Chlorosis" is a term which in itself suggests chromatic changes of the skin and abnormal menstruation. "Anatomical observations," says Virchow, "indicate that the foundations of the chlorotic ailment are very early laid, for . . . the sexual organs are frequently very imperfectly developed." Unsatisfied or perverted sexual instincts also, at

* "In these mammals, the teats of the female are found in the marsupium or pouch. In many respects they are allied to the oviparous vertebrata. In the female, the marsupial bones assist in producing compression of the mammary gland, which is necessary for the alimentation of a peculiarly feeble offspring; and they defend the abdominal viscera from the pressure of the young, as they increase in size during their marsupial existence; and still more when they return to the pouch for temporary shelter. In males, these bones are subservient to the reproductive act. These mammals are also aplacental, and their period of gestation is but thirty-nine days. (In the Virginia opossum, which, however, is destitute of the marsupium, it is twenty-nine.) The young are produced in such an immature state, that earlier observers believed they originated like buds, from the nipples to which they saw them attached. The appearance presented by a young kangaroo of one of the largest species, within twelve hours of its being deposited in the pouch, is described by Prof. Owen, from personal observation in the Zoological Gardens, as follows: 'It resembled an earthworm in the color and semi-transparency of its integument, adhered firmly to the point of the nipple, breathed strongly but slowly, and moved its fore-legs when disturbed. The body was bent upon the abdomen, its short tail tucked in between the hind legs, which were one-third shorter than the fore-legs. The whole length from the nose to the end of the tail, when stretched out, did not exceed one inch and two lines. The mother apparently employs her mouth in placing the young at the nipple, where it remains suspended, involuntarily absorbing milk for a considerable time (probably two months on an average) after which for some months it sucks spontaneously. Although from the first it is enabled, by the muscular power of its lips, to adhere firmly to the nipple, it does not possess strength to obtain the milk by the ordinary process of suckling. In this process it is assisted by the adaptation of a muscle to the mammary gland, which, by contracting, injects the milk from the nipple into the mouth of the adherent fœtus.'"

the puberal period of life, are betrayed by familiar external appearances.

There is some reason to believe that the pigmentary deposit in the skin is more intimately connected with the phases of generation than any other constituent of the epidermis. The extent of variability in the number, and shade of color, of pigment granules, probably exceeds that of any component of the cutis. It is a matter of common observation, even among the negroes of our own country, that exceedingly dark individuals have unusually developed genitals, and that although the race in America has been greatly modified by intermarriage and acclimatization. Few who have observed the negroes in Africa (and my own experience extends to those who are habitually naked on the Coast of Guinea, as well as the Fellahs and Fellahines of lower Egypt,) can have failed to notice this fact. With them, the mamma is apparently destitute of the globular form common in the Caucassian races. But this is an apparent difference only. At puberty, a period which in these dark-skinned races is determined at a very early age, the gland is not unlike that of the white race. Subsequently, it elongates to such an extent, that it is possible for a mother to suckle her infant while it is supported on her back. The negroes are known to be inordinately erotic. Whether they are correspondingly prolific, cannot perhaps be, at this time, determined by statistics. But that they do not enjoy corresponding constitutional vigor, may well be doubted, in view of their extreme predisposition to tuberculosis, scrofula and rickets.

Now, if it can be shown that a congenital or acquired *absence* of pigment granules in the skin, is a concomitant of impotence or sterility, the relation of the rete mucosum to the genital organism can be more clearly detected.

The study of albinism offers such a field for observation. The skin of the pure albino is of a dull, milky-white color. His hair is exceedingly fine, and of a clear white or dirty-yellowish hue. His entire body is covered with a white down. The fundus of his eye is brilliantly red, and its iris is either red or completely transparent. His constitution is delicate; his temperament lymphatic. The depression of the forehead, the flattening of the nose, the prominence of the cheeks, and the woolly character of the hair, as well as an African nativity, usually justify the appellation of "white negro,"

which he has received, and give him the peculiar stamp of an African physiognomy.

The anatomist Buzzi, who has carefully studied these phenomena, declares that he has never found in complete albinism, any trace of the corpus mucosum, nor any pigment granules in the hair. Maurice Raynaud, in a monograph on this subject, remarks that retarded menstruation and sterility are said to be common in female albinoes. "In fact," he proceeds to state, "it does not appear that we possess a well-authenticated case of a child born of a father and mother both albinoes; but there are several recorded cases, where one or both of the parents presented this peculiarity. Ichthyosis was discovered in four of five albinoes examined by Rochas, and in others a corresponding tendency to cutaneous disorders.

Melanopathia, or Addison's disease, is an affection characterized by abnormal coloring of the cutaneous surface. It is perhaps true that its investigation has not yet extended to that point where we can rely upon deductions from its statistics; but it is worthy of remark, that while most observers have had their attention closely directed to the connection between the pigmentary changes and the lesions of the supra-renal capsule, they have not failed incidentally—and perhaps unwittingly—to record a correspondence in derangements of the organs of generation. In the London *Lancet*, for October, 1870, Mr. Houghton reports a case of Addison's disease in a girl seventeen years old, having a tawny skin and herpetic pustules on the face. She had never menstruated, her breasts were undeveloped, her nipples small, and their areolæ quite brown.

Subjoined is a table of sixteen cases of this disease, out of 185 collected in a monograph by Dr. Jaccoud, in each of which it appears that some disorder of the generative organs co-existed with the bronzing of the skin. In all of these cases there was more or less disorganization of the supra-renal capsules—in the last four, however, the cutaneous lesion was not evident. It should be added, as worthy of note, that in 67 of 107 of the cases collected by this author, the supra-renal glands were only secondarily affected.

| No. | Antecedent and Post-mortem History. | Sex. | Age. |
|---|---|---|---|
| 11 | Cancer of uterus, | F. | 28 |
| 12 | Double ovarian dropsy, | F. | 48 |
| 35 | Retraction of testis, | M. | 43 |
| 41 | Dystocia and perineal laceration, | F. | 37 |
| 36 | Syphilis, | M. | 32 |
| 66 | Suppression of menses, | F. | 16 |
| 75 | Uterine tormina, | F. | 47 |
| 90 | Purulent catarrh of Fallopian tubes, | F. | (?) |
| 94 | Excessive post-partum hemorrhage, | F. | 40 |
| 98 | Tumor of right testicle, | M. | 33 |
| 118 | Suppression of menses and ovarian dropsy, | F. | 19 |
| 127 | Entire surface covered with short black hair, extreme development of external genitalia, | F. | 3 |
| 2 | Cancer commencing in clitoris, | F. | 81 |
| 3 | Eclampsia, confinement, and death in three days, | F. | (?) |
| 18 | Cancer of uterus, | F. | (?) |
| 45 | Sudden death after confinement, at term, | F. | (?) |

Of these 107 cases the greater number suffered between thirty and forty years of age—a period of great sexual activity.

There is no standard by which the relative depression of the vital forces in disease can be estimated. The pulse may be registered by a sphygmograph—the temperature, by a thermometer—but it cannot be discovered whether the virile power, which is usually held in abeyance in pathological conditions, is normally quiescent, diminished, or suspended. But in convalescence, we have a qualified opportunity for such investigation. Are evidences of a restoration of this faculty more common in convalescence from disorders having cutaneous lesions, than in others? It is well known that many such lesions, originating from constitutional causes, become, after extinction of such causes, grave maladies of themselves. There are few morbid changes in the skin, where the whole surface is so generally involved, as in yellow fever. I have seen patients afflicted with this disease, whose skin assumed, not a yellow hue, but a color suggestive of incipient gangrene. Now in convalescence from yellow fever the proofs of an unusual and excessive awakening of the venereal appetite, are too numerous to be disregarded. La Roche, who has exhaustively described this disease, refers to this fact, as well as other authorities, who also agree with him in the belief that in latitudes where liability to epidemics of the scourge exist, no one of its predisponents equals in importance excessive sexual indulgence.

Thus far, those cutaneous disorders have been cited which implicate the whole, or very extensive areas, of the integumentary surface. But the etiology of most of the other eruptive diseases has been arranged by dermatologists to include derangements of the generative organs. Among these eruptions may be mentioned —erythema, (both E. fugax, E. laeve, E. intertrigo, and E. tuberosum), as well as the varieties of erysipelas, roseola, urticaria, prurigo, eczema, impetigo, ecthyma and pemphigus. Nor are the exanthematous fevers exempt from complications having regard to the same organs. "An attack of measles," says the late Dr. Trousseau, "is frequently the cause of gangrene of the vulva and vagina in female children—in fact, vulval excoriation may serve as a door of entrance for the malady." Scarlatina is not an infrequent cause of abortion; and the same may be said of variola—the abortion, when occurring, being generally isochronous with the appearance of the pustules.

In a discussion of the subject of acne, before the New York Academy of Medicine, November 7th, 1872, Dr. Weisse took the ground that as the sebacious follicle is but an appendage of the hair follicle, and the sebum matures pomatum for the hair, the coincident activity in the development of hair and the secretion of sebum, is readily appreciated. From eighteen to twenty-five is the period during which occurred, probably, three-fourths of all cases of "folliculitis seboforæ;" and it is more common in males on account of the growth of the beard at this period. Dr. Peters thought it was connected with uterine and ovarian derangement, and that pubescence was a common coincident condition. Dr. Keyes considered acne in the young to be often a signal of sexual distress, of unqualified or perverted sexual yearning, perhaps often unrecognized by the patients; and while he believed in a connection between this cutaneous affection and certain uterine derangements, functional or otherwise, yet neither of these conditions was necessarily connected with the other. As regards treatment, he believed in the curative power of a well-regulated sexual hygiene. Dr. Robert W. Taylor was skeptical as to the origin of acne in uterine derangements; and did not believe that uterine catarrh, or flexions of the uterus, had any influence in their causation; nor had he been able, though he had studied the matter carefully, to make up his mind that such connection existed. Dr. James L. Brown expressed a similar opinion. The president stated that, if an unmarried woman, between twenty and thirty years of age, should consult him for relief of acne, he would inquire into the state of the bowels and kidneys, before inquiring about any uterine derangement; and then he would ascertain if the menstruative secretion was normal. In the case of a married woman, affected with acne, he would first find out whether there was uterine irregularity or ulceration. He thought there was a direct connection between acne and these disorders, although it was not sometimes very evident. The eruption about the mouth, mentioned by Dr. Taylor, was, perhaps, more marked in uterine affections.

These opinions are given to show the wide difference of belief on this subject, among members of the profession eminent for an acquaintance with it. In my own experience one decided case has corroborated my own belief in the causation of the disease under discussion.

Mrs. S—, the mother of one child after eight years of married life, applied to me for the relief of confirmed acne rosacea of the face, of over two years duration. Her appearance was so unsightly that it had almost isolated her from the society in which she had once moved. She had previously resorted to the iron-magnetic springs of Michigan, with some benefit, but the disease had recurred with great severity. For a period of four months I tried the polypharmacy advised by various authorities, and was convinced more than ever, after failure of all treatment, that the disease had been justly named the "reproach of medicine."

Dr. Horatio L. Storer had lately called the attention of the profession to the disastrous results to women of the various means used to prevent conception. Suspecting, finally, that such a cause might obtain in this case, I discovered by questioning that, immediately after insemination, she had recourse to vaginal injections of cold water. On examination, I discovered moderate cervical engorgement. On the strength of my representations she completely abandoned the practice, and in ten months thereafter I delivered her of a healthy female child. The acne disappeared gradually without further medication, and the patient returned to that society from which she had been debarred by her ailment.

Syphilis is a disease which affords a striking example of the connection between the integumentary and generative organs. It effects its entrance to the system by way of the latter—it expends the maximum of its force upon the former. While all the organs of animal life are subject to its destructive agencies it rarely affects the organic viscera. And such affection, if it occur, is peculiar to the later phases of the malady, when it is modified greatly. As a tertiary form, it is subject to different influences, and moderated or entirely removed by different medicaments, from those available in its secondary manifestations.

In conclusion, it may be remarked that however difficult it may appear to be to account for the affinities heretofore suggested, a link of connection may be found in the scope of two great general influences—the hæmopathic and the neuropathic. The alteration of the maternal blood in pregnancy by the admixture, for the first time, of its corpuscles with those secreted from the walls of fœtal vessels, must be exceedingly important; and the suffusion of the vascular elements of the cutis, at all times, with the vital fluid, exposes a large amount to external influences. The genitals and the skin, again, have unsurpassed nervous connections with the cerebral and ganglionic centres. The alteration of the circulation in the cheek during the act of blushing, as well as the change in the position of erectile genitalia, occur under the stimulus of mental and nervous impressions. Such phenomena may be said to be "excito-sensori-reflex."

Floriculturists may be said to demonstrate the fact of a law of compensation, to which these organs are subject. They assert that interference with the rootlets of a plant, while it is young, stimulates at once the reproductive function; as if the individual hastened to reproduce its kind, in anticipation of premature death. And further, that the artificial production of colors in the corolla of flowers, is effected only in accordance with the laws which regulate the generative organism of plants.

In view of all that precedes, it may be concluded that certain physiological and pathological relations, as stated at the outset of these remarks, exist between the generative and integumentary structures; that that assembly of reproductive organs, which is assimilated by function and development to those of animal life, has a more intimate connection with the external surface than that organ which displays the affinities of the vegetative viscera; and that a knowledge of the condition of one series of correlated phenomena, is essential to a correct appreciation of the other.

